# Evaluating non potable groundwater quality in estuarine Islands of Karnataka using GIS and WQI

**DOI:** 10.1038/s41598-025-17682-y

**Published:** 2025-09-24

**Authors:** Sanjana S. Shetty, Shantharam Patil

**Affiliations:** Manipal School of Architecture and Planning, Manipal Academy of Higher Education, Manipal, Karnataka India

**Keywords:** Estuarine islands, GIS, Groundwater, Seasonal variations, Water quality index, Weighted arithmetic water quality index method, Environmental sciences, Hydrology, Health care, Engineering

## Abstract

Fresh groundwater is scarce on estuarine islands due to seasonal fluctuations in the groundwater table and tidal influence. Larger inhabited islands face increasing concerns over access to safe water for household activities. This study evaluates the groundwater quality on Mudukudru Island, one of the largest in the estuary of the Swarna-Sita River system in Udupi district, Karnataka. A total of 43 wells used for non-potable domestic purposes were analyzed during the pre-monsoon season (2021–2022) using a Hanna HI9829 multiparameter testing kit. The Weighted Arithmetic Water Quality Index Method was applied to assess water quality based on four physical parameters (Total Dissolved Solids, Electrical Conductivity, Salinity, Temperature), three chemical parameters (pH, Oxygen Reduction Potential, Dissolved Oxygen), and one biological parameter (Most Probable Number of coliform bacteria). GIS-based spatial maps were developed to visualize distribution patterns. The overall WQI results indicate that approximately 30% of the island’s groundwater falls under the “unsuitable” category. Traces of coliform bacteria were recorded in all samples, indicating that the groundwater is not suitable for drinking without treatment. The findings underscore the need for water purification, regular monitoring, and sustainable management of groundwater used for non-potable household use.

## Introduction

Water sustains life, and life without water cannot be comprehended. Water is a valuable resource, found on the surface or beneath the ground, and the primary source for both is rain. The erratic nature of rainfall and excessive exploitation of surface water have increased the pressure on groundwater. The temporal nature of groundwater distribution varies spatially and is non-uniform, leading to some areas having abundant water and others experiencing deficits due to overuse, seasonal variation, and climate change-induced shifts in recharge patterns^1,2^. Since the 1970s, in India, the dependency on groundwater irrigation has started and has grown in the name of rapid development^3^.

Urbanization and a growing population have severely impacted domestic water demand and access to quality water. This scenario has brought the onus on natural aquifers, leading to water scarcity in many regions across the country^4^. As per the reports from the Central Ground Water Board, GOI, around 60% of irrigated land in the country is dependent on Groundwater irrigation, and 85% of rural drinking water demand is also catered by groundwater sources^3^.

**Fig. 1:**
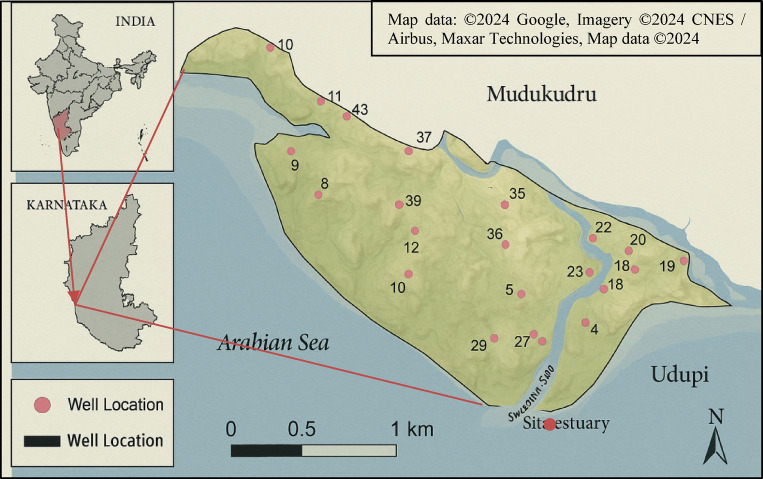
Location map of Mudukudru Island in the Swarna–Sita River estuary, Udupi District, Karnataka, India. The map indicates the 43 sampling well locations along with the surrounding water bodies, the Arabian Sea, major landmarks including Udupi town. Source: Map data: ©2024 Google, Imagery ©2024 CNES / Airbus, Maxar Technologies, Map data ©2024

Waterborne Groundwater quality on estuarine islands is constantly changing because of tidal influences, seasonal variations, dissolved salts that have been leached, water depth, and the subsurface environment^5^. Most of the island’s soil is made up of unconsolidated sediments, which allow contaminants to seep into the soil and have an immediate impact on the aquifers through surface runoff^6^. Human health is associated with the quality of drinking water; about 80% of ailments in humans are waterborne^7^.

Excessive use of groundwater and poor sewage waste disposal into groundwater sources are two anthropogenic activities that harm the quality and availability of groundwater^8^. Water sources, once polluted, are extremely difficult to revive. Therefore, timely monitoring of the physicochemical water quality parameters is crucial for assessing the water environment, ecosystem, hydrochemistry, and ecology, as well as for enhancing water quality^9^. There are numerous techniques for evaluating the quality of groundwater. It is assessed based on physical, chemical, and biological parameters, and the suitability for human use or consumption is deciphered^10^. The most common technique is the Water Quality Index (WQI) method. The Water Quality Index enables a broad investigation of water quality on many levels that impact on a stream’s capacity to support life and determines whether the general state of water bodies poses a possible danger to various uses of water^11^. Derived from the weighted arithmetic method^12^WQI is a dimensionless number that weighs and rates different water quality parameters of a sample and rates them based on overall quality, viz., 0 being excellent and values greater than 100 being unsuitable^13^.

In the current scenario, Geographic Information System (GIS) tools using the kriging interpolation technique and statistical approach have proven fast and effective in the assessment of groundwater quality^14^. It is an efficient and cost-effective method for translating large data sets into projections and maps of spatial distribution that show patterns, relationships, and origins of toxins and pollution.

Recent studies highlight that coastal and estuarine aquifers are especially vulnerable due to the combined impacts of rapid urbanization, agricultural runoff, sea-level rise, and inadequate wastewater management, leading to increasing salinity and biological contamination risks^15,16^. Recent studies emphasize that groundwater in coastal aquifers is increasingly threatened by heavy metal contamination and rising salinity due to both anthropogenic activities and climate-induced changes^17^. Climate variability further compounds this problem by altering recharge rates and contributing to seasonal water stress^18^. Furthermore, the use of GIS and WQI in combination is being increasingly recognized as a robust approach to identify contamination hotspots and support spatial planning for groundwater management^17^. GIS-based approaches combined with hydro chemical analysis have proven effective in delineating vulnerable zones and assessing spatial variability of contaminants in peri-urban and coastal groundwater systems^15,17^. Therefore, the purpose of the study is to ascertain the WQI of groundwater using a Geostatistical approach and assess its appropriateness for household use in the study region.

## Materials and methods

### Description of the study area

Mudukudru is a river island, located in the estuary of the Swarna-Sita composite river system in the Udupi district of Karnataka. The island is the largest among the 15 islands in the estuary, with a total area of 632524 square meters and a perimeter of 4.75 kilometers. The study area is governed by the Kallianpura Gram Panchayat and spans between 13°24‟21.46” N, and 74°43‟35.01” E [6]. The region receives maximum rainfall from July to September. It experiences 182.9 wet days (50.11% of the time) and 384.89 millimeters (15.15 inches) of precipitation each year. In the year 2022, the region received a record of 3762 millimeters of rainfall in September. During the rainy season, it experiences minor to moderate flooding. January is the coldest month with a temperature of 20° C, and the hottest month is May, with a high of up to 38° C. The soil cover of the island is dominated by loamy soil. There are 296 individuals living on the island, with 112 of them being houses, with fishermen making up most of the population. Wells and public water systems are some of the island’s household water sources. A total of 43 wells were selected for sampling based on three criteria: (i) spatial distribution across the island to capture hydrogeological variability, (ii) active domestic usage by households, ensuring that the wells represented water consumed for daily purposes, and (iii) accessibility with informed consent from household members and local authorities. This ensured that the sampling network was both scientifically representative and socially inclusive. The GPS coordinates of each well were recorded, and their locations are shown in Fig. 1. The island predominantly sits on a shallow, unconfined aquifer system, consisting of highly permeable alluvial and lateritic sediments. These sediments allow for rapid infiltration of surface runoff and leaching of contaminants into the groundwater. The aquifer is highly susceptible to contamination due to the unconsolidated sandy-loamy soil, low elevation, and proximity to tidal zones. Seasonal fluctuations in the water table are pronounced, with lower levels observed during the pre-monsoon period. Recharge primarily occurs through rainfall infiltration during the monsoon months. No formal water supply network exists on the island; instead, households rely on individual shallow wells for their daily domestic water needs. Based on household surveys conducted in conjunction with fieldwork, the average water consumption per capita was estimated to be approximately 60–80 L/day, which is below the national rural average^18^.

### Sampling techniques and sampling size

The main purpose of the study is to determine the class of all forty-three samples using the weighted arithmetic index method^12^ and generate spatial maps for those parameters using GIS. The parameters such as pH, Dissolved Oxygen (DO), Electrical Conductivity (EC), Oxygen Reduction Potential (ORP), Total Dissolved Solids (TDS), Salinity (S), Temperature (T), and Most Probable Number (MPN) of Coliform bacteria were taken for assessment. The standards considered for the evaluation of water quality were the Bureau of Indian Standards for Drinking Water Specification IS 10,500^18^, the World Health Organization^7^and the Indian Council of Medical Research^19^ and Central Pollution Control Board^20^ were used while determining the suggested standard values for the WQI calculation. The classification of water quality based on the Weighted Arithmetic WQI method is presented in Table 3.

**Fig. 2:**
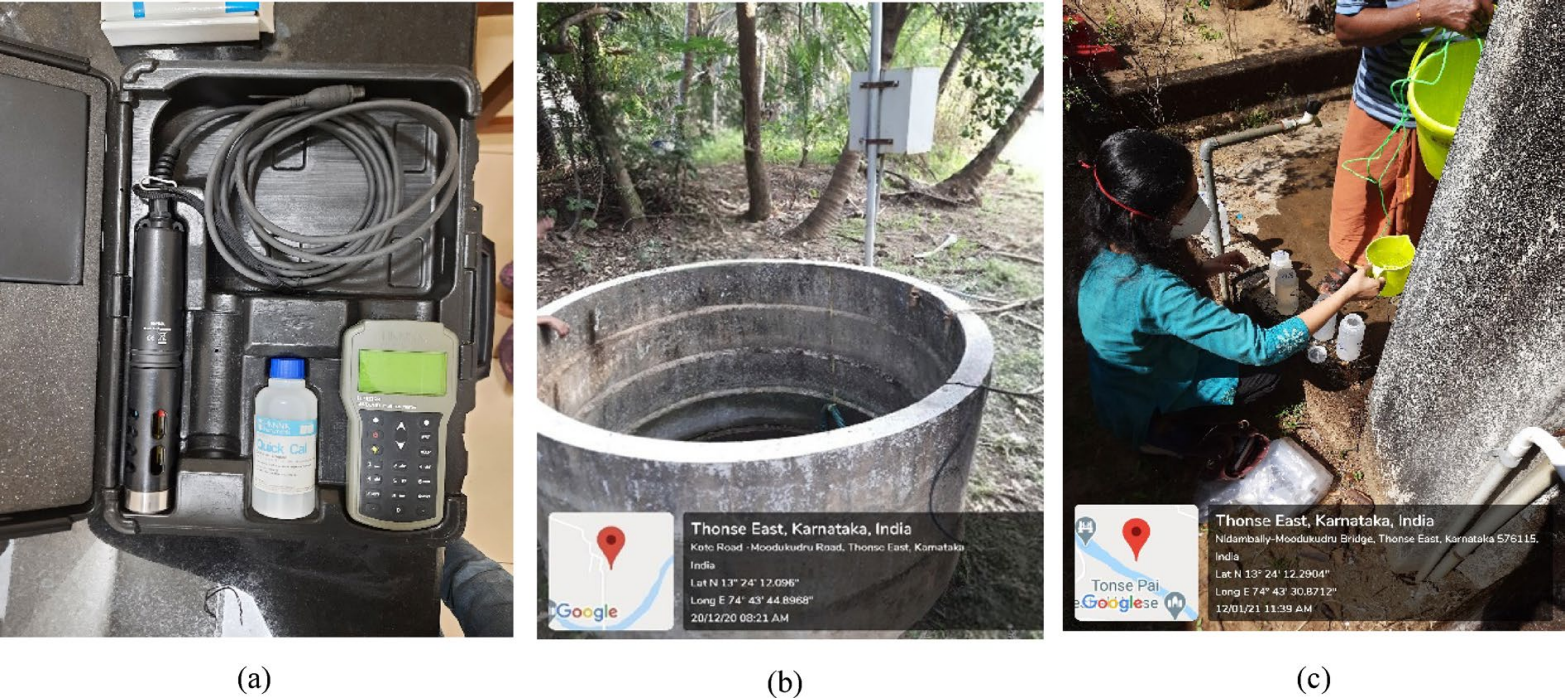
(**a**) Hanna HI9829 multiparameter testing kit used for on-site analysis of physical and chemical parameters; (**b**) on-site testing being conducted at Well-2; (**c**) collection of groundwater samples in a sterile bottle at Well-29 for laboratory-based microbiological analysis. Source: Map data: ©2024 Google, Imagery ©2024 CNES/Airbus, Maxar Technologies, Map data ©2024

The well positions were marked using the Global Positioning System (GPS), the same is represented in Fig. 1. On-site analysis using Hanna HI9829 multiparameter testing kit (Fig. 2a and b) was conducted at the marked 43 sampling points in the study area for checking the physical parameters like TDS, Conductivity, Salinity, Temperature, and chemical parameters like pH, DO, and ORP. The choice of this multiparameter kit was guided by its ability to provide rapid and reliable in-situ measurements, which minimized the risk of sample alteration during transport and ensured accurate representation of groundwater conditions in the estuarine island environment. Given the logistical constraints of the study area, the kit allowed simultaneous testing of multiple parameters with high precision, making it an efficient and validated approach for groundwater quality assessment.

Groundwater samples from 43 wells were collected in sterilized bottles from various sites across the island by following the instructions in IS 1622^18^ to check the biological parameters of MPN. All the samples were properly labeled, indicating the source, time, and date of the collection (Fig. 2c). The biological parameter, MPN of Coliform bacteria, was tested in the laboratory for total coliform (TC), following the standard method stated by the American Public Health Association^21^. While laboratory-based protocols were essential for microbial analysis, on-site testing using the multiparameter kit was prioritized for physicochemical parameters. This approach minimized potential errors arising from sample storage and transport, provided time-efficient results in a remote estuarine setting, and aligned with recent studies validating the robustness of multiparameter kits for groundwater monitoring. Thus, a combination of field-based and laboratory-based methods ensured reliability and practicality within the study context. Table 1 demonstrates the statistical analysis of groundwater. Table 2 depicts a correlation matrix that has been plotted between the groundwater quality parameters.

**Fig. 3:**
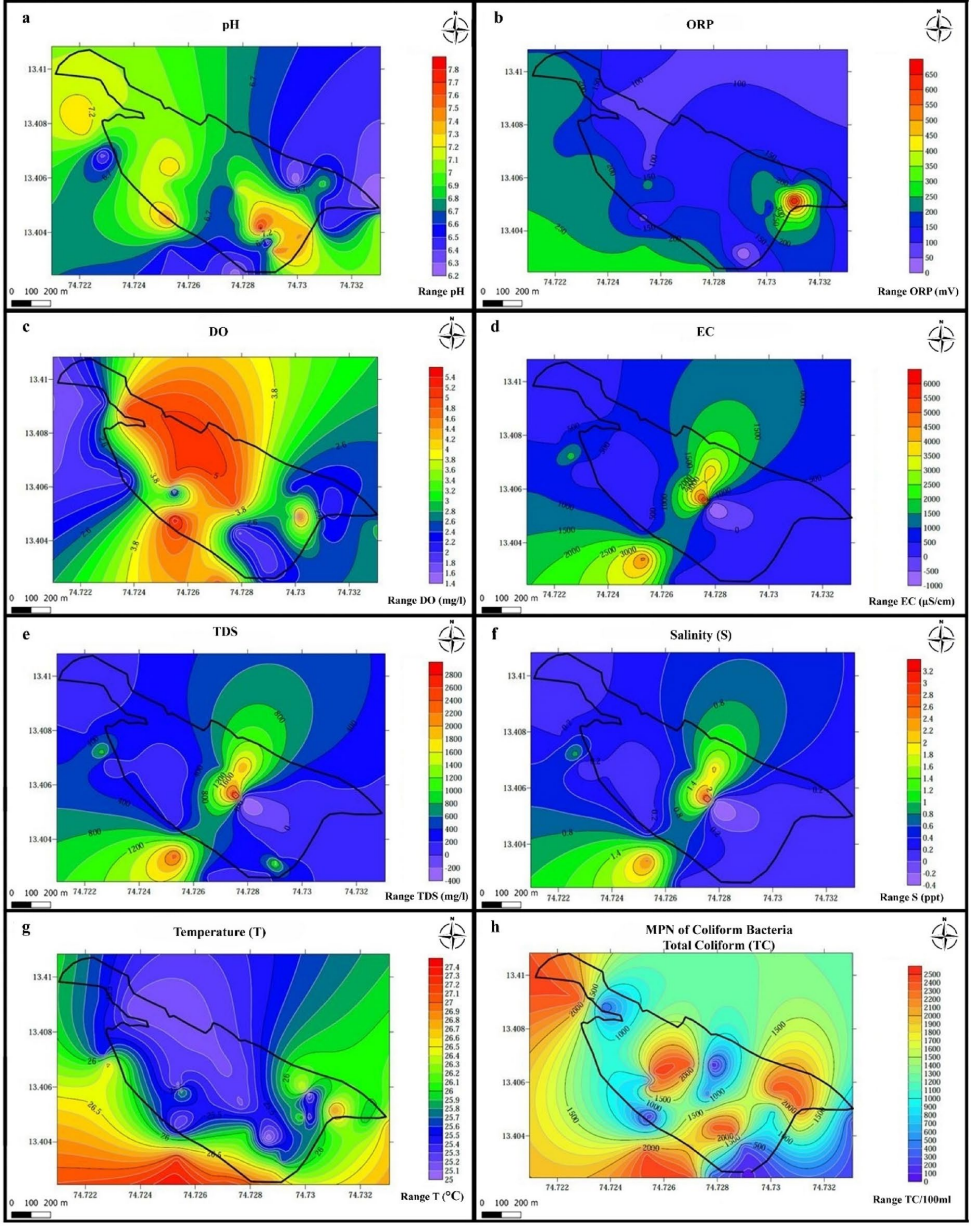
Spatial distribution maps of groundwater quality parameters across Mudukudru Island generated using GIS Kriging interpolation: (**a**) pH; (**b**) Oxidation Reduction Potential (ORP); (**c**) Dissolved Oxygen (DO); (**d**) Electrical Conductivity (EC); (**e**) Total Dissolved Solids (TDS); (**f**) Salinity; (**g**) Temperature; and (**h**) Most Probable Number (MPN) of coliform bacteria. *The interpolation extends slightly beyond the island boundary for spatial continuity and visualization; the analysis and interpretation are confined strictly within the delineated island perimeter. The black outline represents the official boundary of Mudukudru island.

The results for each parameter have been spatially depicted using GIS software. The Water Quality Index (WQI) was calculated using the weighted arithmetic method based on eight selected water quality parameters, as shown in Table 4. The Kriging interpolation technique is used for this study, as it gives a realistic picture of the phenomenon happening around the sampling points. It is based on a geostatistical model that includes autocorrelation, which checks the statistical correlation between the measured points and creates a predictive surface around the point with some measure of certainty and accuracy of the predictions^22^. A spatial distribution map of the eight water quality parameters is plotted using ArcGIS Desktop, Version 10.1 (Esri, Redlands, CA, USA), available at https://www.esri.com/en-us/arcgis/products/arcgis-desktop/overview^23^, as shown in Fig. 3.

No experimental interventions were conducted on human subjects. Ethical clearance was taken from Kasturba Medical College and Kasturba Hospital Institutional Ethics Committee (Registration No. ECR/146/Inst/KA/2013/RR-19) with certificate no.IEC:135/2021, for the collection of water samples from households and their microbial analysis. Informed consent was taken from local authorities and head members representing individual households for collecting water samples.

**Table 1:** Statistical analysis of analyzed physicochemical groundwater quality parameters. Highlighted well numbers indicate excellent and good quality water along with MPN value (< 500) as specified by CPCB, 1979.

Samples	Physical parameters				Chemical parameters			Biological parameters
	TDS (mg/l)	EC (µS/cm)	Salinity (ppt)	Temperature (°C)	pH	DO (mg/l)	ORP (mV)	MPN of Coliform bacteria/100 ml
**Well-1**	88	176	0.08	26.73	6.64	2.13	170.3	**140**
**Well-2**	1199	400	0.19	26.22	6.81	1.8	-3	**350**
Well-3	142	283	0.13	25.59	7.57	1.94	39.7	540
**Well-4**	133	266	0.13	25.78	6.31	1.85	142.7	**280**
Well-5	136	272	0.13	24.97	7.78	2.06	122	2400
Well-6	292	585	0.28	26.03	6.75	1.71	173.4	2400
Well-7	52	108	0.05	26.57	6.33	2.07	201.7	920
Well-8	953	1906	0.97	25.49	6.82	2.33	167	180
Well-9	216	431	0.21	25.68	7.28	2.1	196.7	920
Well-10	65	130	0.06	25.96	7.07	1.74	197.2	2400
Well-11	114	227	0.11	25.53	6.95	2.02	243.4	2400
**Well-12**	229	458	0.22	25.91	7	1.96	242.8	**240**
Well-13	138	276	0.13	25.88	6.9	1.92	235.7	2400
**Well-14**	45	90	0.04	26.7	7.12	2.88	124.9	**240**
**Well-15**	54	109	0.05	26.42	6.24	2.46	163.6	**220**
Well-16	164	328	0.16	25.93	6.28	2.53	133.5	540
**Well-17**	64	128	0.06	25.9	6.45	2.47	151.4	**350**
Well-18	156	313	0.15	26.9	6.49	2.02	682	2400
**Well-19**	149	298	0.14	27.03	6.48	2.18	99.9	**350**
**Well-20**	183	366	0.17	26.02	6.87	2.25	149	**240**
Well-21	62	124	0.06	25.1	6.65	2.76	207.6	2400
**Well-22**	92	184	0.09	26.49	6.18	2.19	245.2	**350**
Well-23	65	129	0.06	26.09	7.15	3.08	234.3	920
**Well-24**	96	193	0.09	25.89	7.39	3.19	256	**350**
Well-25	75	149	0.07	26.12	6.4	2.35	276.2	1600
**Well-26**	141	281	0.13	27.08	6.24	4.2	269.1	**240**
Well-27	109	217	0.1	27.18	6.44	4.73	260.3	2400
Well-28	125	250	0.12	27.07	6.51	4.28	260.7	2400
Well-29	2373	4750	2.53	27.2	6.49	4.58	261	2400
Well-30	197	393	0.19	25.18	7.06	4.93	97.8	920
Well-31	102	204	0.1	25.22	7.03	5.12	118.7	920
Well-32	209	4.18	0.2	25.18	6.73	4.92	125.6	920
Well-33	59	118	0.05	25.25	7.18	4.88	107.4	920
Well-34	3378	6757	3.7	25.19	6.68	4.76	119.1	350
Well-35	593	1184	0.59	25.18	6.77	5.12	136.9	2400
Well-36	2003	4003	2.12	25.27	6.82	4.82	128.8	49
**Well-37**	67	134	0.06	24.64	7.02	2.44	203.6	**240**
Well-38	78	156	0.07	25.34	7.26	4.96	91.7	2400
**Well-39**	155	310	0.15	25.13	7.2	4.52	115.6	**350**
**Well-40**	90	181	0.08	25.25	7.17	5.34	128.5	**240**
**Well-41**	179	357	0.17	25.21	7.04	5.29	111.6	**240**
**Well-42**	141	277	0.13	25.27	7.49	4.92	78.2	**350**
**Well-43**	240	480	0.23	25.11	7.1	5.02	95	**350**
Maximum	3378	6757	3.7	27.2	7.78	5.34	682	2400
Minimum	45	4.18	0.04	24.64	6.18	1.71	-3	49
Mean	353.51163	650.8181395	0.3383721	25.85767442	6.840465	3.274884	175.8791	1014.395349
Std. deviation	674.51021	1327.090442	0.7179466	0.700896831	0.393022	1.335482	103.5199	921.0241489

**Table 2:** Correlation matrix between various groundwater quality parameters.

Parameters	pH	ORP	DO	EC	TDS	Salinity	T	MPN
pH	1							
ORP	-0.372*	1						
DO	0.172	-0.31*	1					
EC	-0.116	-0.053	0.28	1				
TDS	-0.118	-0.049	0.246	0.973**	1			
Salinity	-0.12	-0.057	0.289	0.999**	0.974**	1		
T	-0.591**	0.503**	-0.298	-0.047	-0.035	-0.054	1	
MPN	0.002	0.376*	-0.04	-0.037	-0.063	-0.039	0.127	1

Bold values reflect a significant correlation among the correlated physicochemical parameters.

*Correlation is significant at the 0.05 level(2-tailed).

**Correlation is significant at the 0.01 level(2-tailed).

### Calculation of water quality index

In the current study, eight parameters (pH, DO, ORP, TDS, EC, Salinity, Temperature, and Coliform bacteria) were selected to calculate the WQI using the weighted arithmetic method^10^. The selection of these eight parameters was guided by three main considerations: (i) their regulatory relevance, as prescribed by the Bureau of Indian Standards (IS 10500:2012), WHO guidelines, and CPCB recommendations; (ii) their sensitivity to estuarine hydrogeological conditions, where salinity intrusion, microbial contamination, and redox changes are dominant concerns; and (iii) practical feasibility, ensuring comprehensive coverage of physical, chemical, and biological indicators within the scope of this study. While additional parameters such as heavy metals and ionic ratios are important, they were beyond the scope of this work and have been identified as recommendations for future research. The classification of water quality based on WQI values is presented in Table 3.

*Stage 1*: Calculation of the unit weight (**W*n***) factor for individual parameters.

$${\text{W}}n = {\text{K}}/{\text{S}}n$$

The unit weight of each parameter was computed by a value inversely proportional to the permissible value recommended by each standard **1/S*n***.

The summation of all the unit weights was calculated (∑1/S*n*). The proportionality constant (K) is calculated by the formula:

$${\text{K}} = \left( {1/\left( {1/\sum 1/Sn} \right)} \right)$$

where S*n* is the Standard permissible value of nth parameter.

*Stage 2*: Sub-Index values calculation.

$${\text{Q}}n = \left( {{\text{V}}n{-}{\text{Vo}}} \right)/\left( {{\text{Sn}} - {\text{Vo}}} \right) \times 100$$

where Q*n* is the Sub-Index Value, V*n* = mean concentration value; Vo = ideal value of nth parameter and Sn is the permissible standard value.

However, the Sub-Index values for pH and DO is calculated differently, i.e.

$${\text{Q}}_{{{\text{pH}}}} = \left[ {\left( {{\text{VpH}} - 7} \right)/\left( {8.5 - 7} \right)} \right] \times 100$$

$${\text{Q}}_{{{\text{DO}}}} = \left[ {\left( {{\text{VDO }}14.6} \right)/\left( {5 - 14.6} \right)} \right] \times 100$$

*Stage 3*: WQI Calculation.

The overall WQI is calculated by combining stage 1 and 2 calculations.

$${\text{WQI}} = \sum {\text{W}}n \times {\text{Q}}n/\sum {\text{W}}n$$

**Table 3:** Classification of water quality and status based on weighted arithmetic WQI Method^10^.

WQI	Rating class
0–25	Excellent
26–50	Good
51–75	Poor
76–100	Very poor
> 100	Unsuitable

**Table 4:**
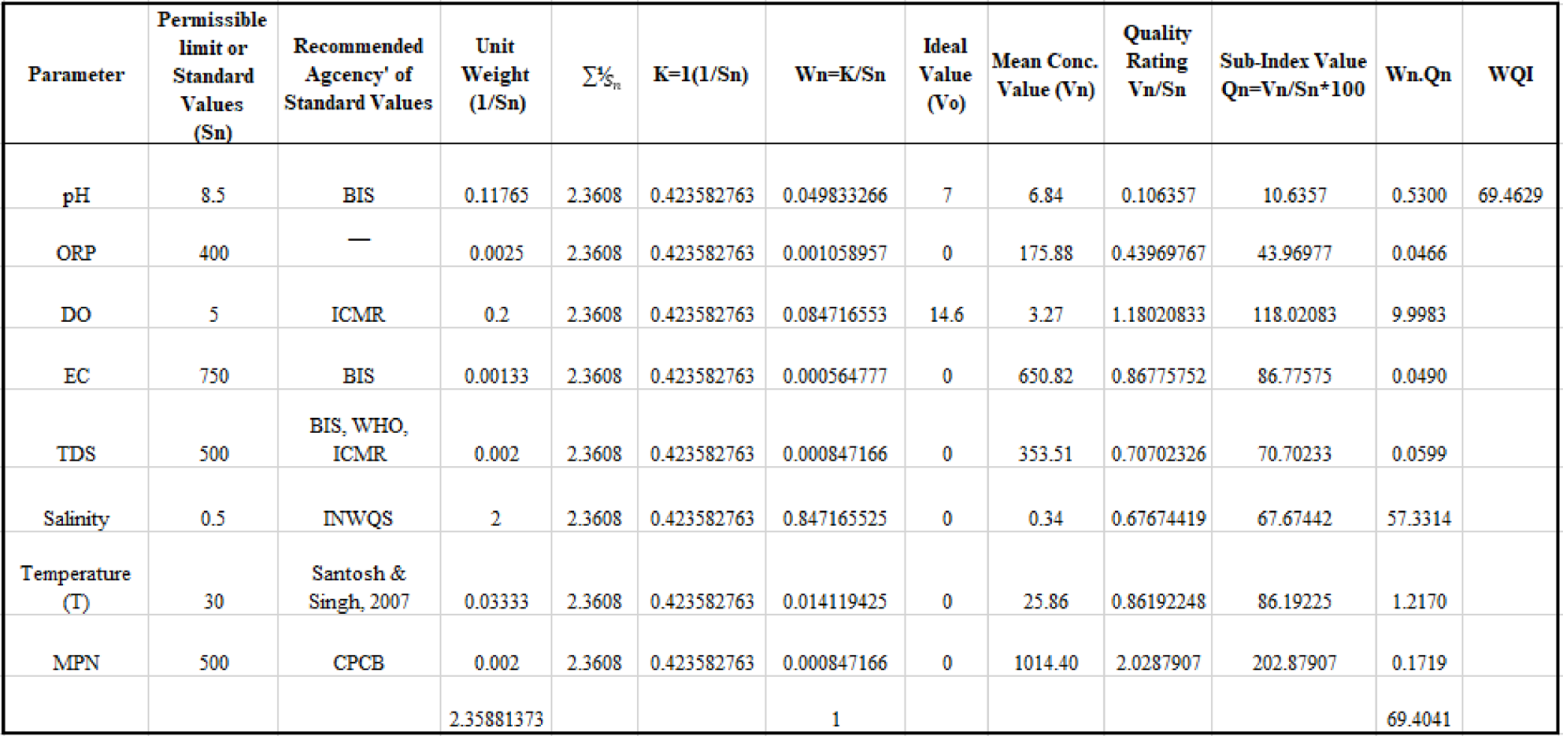
Calculation of WQI using the weighted arithmetic method considering eight water quality parameters.

**Table 5:** Water quality rating of individual wells.

Well water samples	WQI	Based on physicochemical parameters
		Classification as per WQI range
Well-1	27.17	Good
Well-2	45.80	Good
Well-3	36.55	Good
Well-4	37.01	Good
Well-5	37.44	Good
Well-6	61.64	Very Poor
Well-7	23.27	Excellent
Well-8	178.10	Unsuitable
Well-9	49.19	Good
Well-10	23.49	Excellent
Well-11	31.70	Good
Well-12	50.00	Good
Well-13	35.38	Good
Well-14	18.89	Excellent
Well-15	23.09	Excellent
Well-16	41.68	Good
Well-17	24.08	Excellent
Well-18	40.23	Good
Well-19	37.93	Good
Well-20	41.64	Good
Well-21	23.48	Excellent
Well-22	30.39	Excellent
Well-23	22.34	Excellent
Well-24	28.06	Good
Well-25	26.32	Good
Well-26	35.27	Good
Well-27	29.38	Good
Well-28	32.95	Good
Well-29	443.59	Unsuitable
Well-30	42.51	Good
Well-31	26.89	Good
Well-32	44.73	Good
Well-33	19.08	Excellent
Well-34	641.67	Unsuitable
Well-35	111.38	Unsuitable
Well-36	371.88	Unsuitable
Well-37	22.29	Excellent
Well-38	22.94	Excellent
Well-39	36.41	Good
Well-40	23.65	Excellent
Well-41	38.60	Good
Well-42	33.620	Good
Well-43	49.28	Good

## Results

The water quality parameters considered for arriving at the WQI are mentioned in Table 1 and spatially represented using ArcGIS 10.1 software in Fig. 3a–h. In this study, the quality of water is assessed considering four physical, three chemical, and one biological parameter, which are discussed.

### Analysis of statistical values, correlation matrix, and relative weightage

The correlation matrix, general statistical analysis, and relative weightage of the parameters affecting groundwater quality are mentioned in Tables 1 and 2, and 4. The correlation matrix of eight water quality parameters has been analyzed using IBM SPSS Statistics for Windows, Version 26.0 (IBM Corp., Armonk, NY, USA), available at https://www.ibm.com/products/spss-statistics. Table 2 shows the correlation of four parameters, TDS, Salinity, Temperature, and EC, to be highly statistically significant, reflecting more than 0.5 correlation, having a p-value ≤ 0.01. TDS vs. EC, Temperature (T) vs. ORP, Salinity vs. EC, ORP vs. pH, MPN vs. ORP, and DO vs. ORP parameters demonstrate the most pertinent correlation and have a significant influence on the overall evaluation of groundwater quality.

### Spatial distribution pattern

The groundwater quality parameters are spatially represented in the maps shown in Fig. 3a–h. According to the pH, the spatial distribution pattern of groundwater on the island is mainly found to be alkaline in the N-W region of the island. There are a few areas in the S-E region where the pH value indicates the acidic nature of groundwater (Fig. 3a). The S-E region of the island shows healthier water with ORP ranging between 300 and 500 mV, whereas 90% of the island has ORP value below 200 mV, which indicates low Dissolved Oxygen (DO), high nitrites or high Dissolved Organic Carbon (DOC). A high concentration of ORP is noted in Well-18, which is in the S-E region of the island (Fig. 3b). In the study area, the range of DO recorded in the S-E region varies between 1.4 and 3.2 mg/L, which indicates severely polluted water, and towards the N-W, the values majorly range between 3 and 5.34 mg/L, which is classified to be polluted to slightly polluted (Fig. 3c).

The EC is primarily highest (> 750 mg/l) in the N-W and S-E regions, with a few minor isolated spots in the central region (Fig. 3d–e). Similarly, the TDS values correspond to the EC value having < 1000 mg/l in the N-W and S-E region, which fall under the acceptable limits^5^. The presence of Salinity in groundwater is found to be high in the central region, with values exceeding 1 ppt, whereas the rest of the island shows values < 1 ppt, indicating an acceptable limit of salinity in groundwater (Fig. 3f). The temperature distribution map shows that the water temperature throughout the island is found to be within the acceptable range of 25 °C to 27 °C (Fig. 3g). The MPN of Coliform Bacteria is a crucial indicator of water quality, which also indicates the other contributing factors leading to the contamination of the groundwater. The spatial map for MPN recorded on the island shows the S-E and Northern regions have a higher concentration of MPN (> 500 mg/L) and a few scattered patches in the central region show MPN < 500 mg/L. This indicates the influence of anthropogenic and environmental factors leading to the bacterial contamination of water (Fig. 3h).

### Spatial map of water quality index

**Fig. 4:**
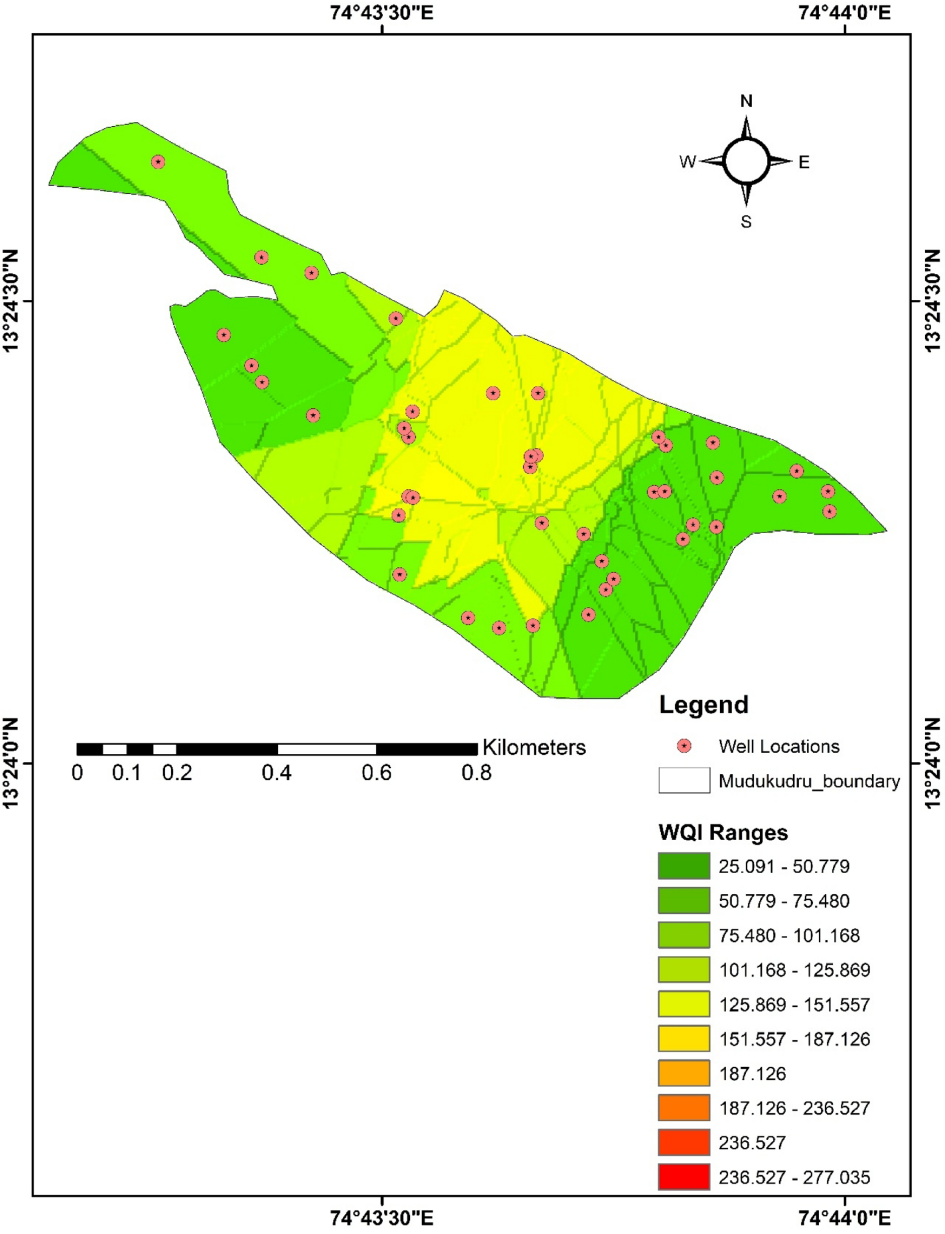
Water Quality Index (WQI) classification map of Mudukudru Island. The wells are categorized into five quality classes—excellent, good, poor, very poor, and unsuitable—based on WQI values.

Using ArcGIS 10.1, the WQI map has been created to represent the various physicochemical quality classifications, such as excellent, good, poor, very poor, and unsuitable, for each well as shown in (Table 5; Fig. 4). The WQI map of the Island shows that most of the groundwater is of good quality (26–50), whereas minor pockets in the N-W and central region have very poor quality (76–100) to unsuitable quality (> 100) (Fig. 4).

## Discussion

The study of the Estuarine Island is essential, as the region experiences fluctuating water quality throughout the year, posing significant challenges to local communities. It has been discovered that public health issues and habitat destruction are inextricably related to water contamination, which can be prevented with timely monitoring of water quality. Recent attention to water quality parameter monitoring and evaluation has shown a direct connection between water contamination, public health issues, and habitat destruction. Timely monitoring can mitigate such concerns. However, many wastewater quality parameters are typically assessed through expensive and time-consuming laboratory techniques, demonstrating a critical need for rapid, on-site monitoring methods^24^.

For this study, the Hanna HI9829 multiparameter testing kit was employed to assess key physicochemical parameters such as TDS, EC, Salinity, Temperature, pH, ORP, and DO. For the on-site evaluation, the Hanna HI9829 multiparameter testing kit was used to assess physicochemical parameters such as TDS, EC, Salinity, Temperature, pH, ORP, and DO. Biological parameters, including Total Coliform (a significant indicator of domestic water quality), were analyzed in the lab. The WQI map was developed based on these physicochemical parameters. It is inferred from the analysis that the overall quality of water on the Island is good. However, it is found that all the wells on the Island have MPN > 0, which makes them unfit for drinking without further treatment. This study raises awareness among island inhabitants about the water quality in their wells, facilitating informed decisions regarding the need for further treatment to ensure safe water consumption.

The scope of the study was limited to the pre-monsoon period; however, it would be beneficial for future research to extend the analysis to other seasons to better understand seasonal variations in water quality. Although this study focused on physicochemical and biological parameters, the assessment of heavy metal contamination, which is crucial for a comprehensive understanding of groundwater health, was beyond its scope. Given that estuarine aquifers are vulnerable to both natural (e.g., saline intrusion) and anthropogenic factors (e.g., agricultural runoff), future studies should include heavy metal profiling—especially for parameters like arsenic, lead, iron, and mercury.

The spatial patterns of contamination observed suggest that multiple sources contribute to the degradation of groundwater quality. The presence of coliform bacteria in all wells points to widespread microbial contamination, likely stemming from inadequate sanitation, open defecation, and poorly managed wastewater systems. The higher levels of EC and salinity observed in specific wells (e.g., Well 34) are likely the result of agricultural runoff, irrigation return flows, and fertilizer leaching. In addition to anthropogenic pressures, climate variability and long-term climate change are also critical factors influencing groundwater quality in estuarine islands. Variability in monsoon rainfall alters recharge rates and leads to seasonal stress on aquifers, while sea-level rise exacerbates saline intrusion into shallow unconfined aquifers. Rising air and water temperatures further influence dissolved oxygen levels and microbial activity, intensifying contamination risks. These processes act synergistically with anthropogenic activities, making estuarine groundwater systems increasingly vulnerable. Recent studies highlight that climate-driven salinization and altered recharge regimes are emerging threats, which reinforces the urgency of integrated management strategies for estuarine aquifers^1,2^. Similar findings have been reported in eastern and coastal India where WQI analysis coupled with multivariate statistics revealed anthropogenic pressures such as intensive farming and sanitation failures as key contamination sources^14^. The island’s unconfined, permeable alluvial soils facilitate rapid contamination of groundwater. The correlations observed between ORP, MPN, and DO reflect biogeochemical interactions within the aquifer, revealing areas of low redox potential and heightened microbial activity. These findings underscore the need for integrated water resource management strategies that address both natural hydrogeological vulnerabilities and anthropogenic pressures on water quality.

## Conclusion

The results of the current study on the estuarine island of Mudukudru indicate that anthropogenic activities have significantly degraded the quality of groundwater. Improper sewerage waste management has been identified as a major contributor to elevated Total Coliform values in groundwater, particularly in the central and south-eastern regions of the island. The contamination is likely exacerbated by the porous nature of the estuarine soils, which facilitates the infiltration of contaminants into the groundwater. Given these findings, there is an urgent need for frequent and systematic water quality monitoring on the island. This ongoing monitoring will help identify and track the sources of contamination and the extent of their impact on groundwater quality, providing crucial data for the development of effective pollution prevention and mitigation strategies tailored to Mudukudru Island’s specific needs.

To create comprehensive pollution prevention programs, it is essential to analyze temporal and spatial trends in water quality. Understanding these patterns will enable stakeholders to pinpoint periods of heightened vulnerability and prioritize areas for immediate intervention. Public awareness and community involvement in monitoring activities will also be vital in ensuring long-term water quality improvements. In summary, addressing the adverse effects of anthropogenic activities on Mudukudru Island’s groundwater requires a multifaceted approach that includes regular monitoring, data analysis, and community engagement. By combining these efforts, we can safeguard the island’s water resources for future generations.

Further studies should include seasonal monitoring and heavy metal analysis to better understand the long-term impacts of both natural and anthropogenic contaminants on groundwater quality in estuarine island environments. While this study did not confirm seawater intrusion due to the absence of chloride or ionic ratio data, the spatial distribution of EC and salinity suggests that such processes may be influencing groundwater quality in specific zones. Future research should incorporate ion-based analyses (e.g., Cl^−^, Na^+^/Ca^2+^ ratios) and seasonal comparisons to assess the extent and progression of seawater intrusion. Moreover, integrating hydrochemical characterization with spatial GIS modeling, especially for detecting heavy metals and climate-driven salinization, would provide a comprehensive understanding of aquifer health in such dynamic estuarine environments^15,16^. Additionally, long-term monitoring of groundwater quality, combined with land-use mapping, will enhance our understanding of anthropogenic impacts and help inform mitigation strategies.

## Data Availability

The datasets used and/or analysed during the current study available from the corresponding author on reasonable request.

## Abbreviations

WQI: Water Quality Index
TDS: Total dissolved solids
EC: Electrical conductivity
S: Salinity
T: Temperature
ORP: Oxygen reduction potential
DO: Dissolved oxygen
MPN: Most Probable Number
